# Exploration of Novel Prognostic Markers in Grade 3 Neuroendocrine Neoplasia

**DOI:** 10.3390/cancers13164232

**Published:** 2021-08-23

**Authors:** Rebecca Abdelmalak, Mark P. Lythgoe, Joanne Evans, Michael Flynn, Justin Waters, Andy Webb, David J. Pinato, Rohini Sharma

**Affiliations:** 1 Division of Surgery and Cancer, Hammersmith Hospital, Imperial College London, Du Cane Road 72, London W12 0HS, UK; rebecca.abdelmalak16@imperial.ac.uk (R.A.); m.lythgoe@imperial.ac.uk (M.P.L.); joanne.evans10@nhs.net (J.E.); david.pinato@imperial.ac.uk (D.J.P.); 2 Department of Medical Oncology, University College London Hospital, London WC1E 6BT, UK; michael.flynn9@nhs.net; 3 Kent Oncology Centre, Maidstone and Tunbridge Wells NHS Trust, Canterbury CT1 3NG, UK; justin.waters@nhs.net; 4 Department of Oncology, Brighton and Sussex University Hospitals, Brighton BN2 5BE, UK; andrew.webb@bsuh.nhs.uk

**Keywords:** prognosis, neuroendocrine tumours, inflammation, survival

## Abstract

**Simple Summary:**

High grade neuroendocrine tumours and carcinomas (NETs/NECs) behave aggressively and have limited survival outcomes. The mainstay of therapy is systemic therapy, in which the associated side-effects are a key consideration in a palliative population. We have conducted a retrospective review of patients with high grade NETs and NECs to determine possible tests that will predict survival before patients start treatment. This will allow patients to avoid potentially toxic treatment that is unlikely to be of benefit.

**Abstract:**

Background: High-grade neuroendocrine tumours and carcinomas (NET/NECs) behave aggressively, typically presenting at an advanced stage. Prognosis is poor, with median survival between 5 and 34 months. The mainstay of treatment is palliative systemic therapy. However, therapy carries a risk of toxicity, which can reduce quality of life. Therefore, accurate prognostic scores for risk stratification of patients with high-grade NET/NECs are needed to help guide patient management to decide whether active treatment is likely to improve overall survival (OS). We aimed to compare the prognostic ability of published prognostic scores to predict OS in a cohort of patients with high-grade NET/NECs of any primary site. Methods: Treatment, biochemical and clinicopathological data were collected retrospectively from 77 patients with high-grade NET/NECs across three hospitals between 2016 and 2020. Variables including performance status (PS), Ki-67, age at diagnosis, previous treatment and presence of liver metastases were recorded. Pre-treatment neutrophil-lymphocyte ratio (NLR), platelet-lymphocyte ratio, modified Glasgow prognostic score (mGPS), and gastrointestinal neuroendocrine carcinoma (GI-NEC) score were derived. Univariable and multivariable survival analyses were used to assess prognostic ability. Results: The median age of the cohort was 63 years (range: 31–85); 53% of subjects were female. Grade 3 NETs (G3-NETs) were identified in 32 patients and NECs in 45 patients. The median OS was 13.45 months (range: 0.87–65.37) with no difference observed between G3-NETs and NECs. Univariable analysis revealed that NLR (*n* = 72, *p* = 0.049), mGPS (*n* = 56, *p* = 0.003), GI-NEC score (*n* = 27, *p* = 0.0007) and Ki-67 (*n* = 66, *p* = 0.007) were significantly associated with OS. Multivariable analysis confirmed that elevated mGPS (*p* = 0.046), GI-NEC score (*p* = 0.036), and Ki-67 (*p* = 0.02) were independently prognostic for reduced OS across the entire cohort. mGPS was identified as an independent prognostic factor in G3-NETs. Independent predictors of OS in NECs were PS and Ki-67. Conclusions: mGPS, PS and Ki-67 are independent prognostic markers in high-grade NET/NEC patients. Our study supports the use of these prognostic scores for risk stratification of patients with high grade cancers and as useful tools to guide treatment decisions.

## 1. Introduction

Neuroendocrine neoplasia (NEN) is relatively rare; however, unlike many other tumour types, the incidence is rising significantly, increasing almost five-fold over 30 years in the United States. The term ‘NEN’ describes a collection of heterogenous neoplasms which arise from the cells of endocrine glands as well as the diffuse neuroendocrine system [1]. The most common primary tumour sites are in the gastrointestinal (GI) tract, including the pancreas (62–67%), and the pulmonary system (22–27%) [1]. The management and prognosis of NENs is guided by tumour stage, grade and morphology. Grade is determined by the mitotic count and/or Ki67 proliferation index: low grade or grade 1: Ki67 < 3%, moderate or grade 2: Ki67 between 3–20%, and high grade or grade 3: Ki67 > 20%. In recognition of the heterogeneity in survival and response to treatment in patients with high grade NENs, grade 3 NENs are further classified into well differentiated neoplasias (G3-NETs) and poorly differentiated carcinomas (NEC) [2,3,4].

The majority of patients with NECs present with metastatic disease and in this setting palliative chemotherapy is the mainstay of treatment [5,6]. Etoposide/platinum-based chemotherapy is the standard first-line combination, borne from its established therapeutic role in small cell lung cancer [7,8] with reported response rates of 31–67% and median OS of 12–19 months [7,8,9]. Irinotecan and the alternative platinum-based chemotherapy, carboplatin, are also used with varying benefit; prospective randomised trials are absent and therefore the superiority of any one type of chemotherapy regimen cannot be established [6]. G3-NETs respond poorly to chemotherapy; recent publications suggest a low response rate to platinum based regimens [10,11]. Guidelines suggest this group of patients may benefit from therapies used in grade 2 NENs but there is a paucity of prospective data [4]. The role of systemic chemotherapy in metastatic NENs is that of palliation, where the primary aim of therapy is to improve quality of life. Given that chemotherapy carries a substantial risk of toxicity, accurately predicting at diagnosis which patient sub-populations are likely to have better prognoses and derive clinical benefit from treatment, in terms of OS, is of key importance. There is also growing evidence of the benefit of peptide receptor radiotherapy in selected patients with G3-NETs [12]. Given the cost and duration of therapy, prognostic indices are required.

The NORDIC-NEC study of 305 patients investigated possible prognostic factors in a mixed cohort of G3-NETs and NECs. The study identified performance status (PS) as the strongest predictor of survival; however, PS is a subjective assessment and has been shown to be limited in predicting patient outcomes [13]. Elevated platelets (>400 × 10^9^/L), lactate dehydrogenase (LDH), primary site and Ki-67 of >55% were all identified as independent prognostic factors of survival [3]. Contrastingly, Lamarca et al. [14] identified a Ki-67 level of 80%, in their design of the gastrointestinal neuroendocrine carcinoma (GI-NEC) score, as an important cut-off for survival between groups. Other significant prognostic markers incorporated into the GI-NEC score were LDH, PS, alkaline phosphatase (ALP) and presence of liver metastases [14]. A further retrospective study of 151 patients with intestinal and pancreatic NENs (70% of whom were grade 3), identified age at diagnosis >65 years, presence of metastases, higher grade and large primary tumour size as adverse prognostic markers [15].

Systemic inflammation has been shown to be prognostic in a large number of tumour types and as a marker of more aggressive tumour behaviour [16]. The modified Glasgow prognostic score (mGPS) incorporates the inflammatory marker C-reactive protein (CRP) and albumin levels, which reflect systemic inflammation, in a well validated score that stratifies patients into three prognostic groups [17]. Other inflammatory markers that are reported as adversely prognostic are platelet-lymphocyte ratio (PLR) ≥ 300 × 10^9^/L and neutrophil-lymphocyte ratio (NLR) ≥ 5 [18]. The prognostic role of the inflammatory scores has been evaluated in well-differentiated NENs, but has not been evaluated in G3-NET/NECs [19]. Therefore, our study aimed to assess and compare the prognostic ability of inflammatory based prognostic scores to GI-NEC score and Ki-67, to predict OS in a mixed cohort of patients with G3-NETs and NECs of any primary site in a real-world population.

## 2. Methods and Materials

### 2.1. Study Design

This was a retrospective multi-center cohort study of patients with G3-NET/NECs, defined as Ki-67 > 20%, attending Imperial College Healthcare NHS Trust, Brighton and Sussex University Hospital, and Maidstone Hospital. Data was collected from available paper and electronic patient records between January 2016 and March 2020.

Patient age, sex, date of diagnosis, primary tumour site, stage, location of metastases, Ki-67, morphology, first and second-line treatments, number of treatment cycles, best response to chemotherapy as determined by the Response Evaluation Criteria in Solid Tumours (RECIST v1.1) [20], baseline haematological biochemical results (before first treatment), PS, date of first chemotherapy treatment, date of radiological progression and date of death or last follow-up were collected where available.

### 2.2. Study Population

Although 84 patients were originally identified, seven were excluded due to inaccessible archived patient records. The remaining 77 patients all had a confirmed G3-NETs or NECs of any primary site or G3-NETs or NECs of unknown primary—excluding paragangliomas and phaeochromocytomas. 

### 2.3. Prognostic Scores

Pre-treatment laboratory tests required to derive the prognostic scores were collected including white blood cell (WBC), neutrophil, lymphocyte, platelet, albumin, CRP and LDH. The derivation of all prognostic scores studied is illustrated in Table 1.

The endpoint was overall survival (OS), defined as the period between initial tissue diagnosis to last clinical follow-up or date of death. OS was chosen as the more appropriate outcome for assessment rather than progression-free survival, as OS data was more complete. Additionally, OS is more commonly reported with prognostic scores within this patient cohort, thereby improving the ease of comparisons to the literature.

Patient data was collected as part of an audit with approval from the Imperial College Healthcare Tissue bank (sub-collection reference number R14014). Patient data was fully anonymised, and any identifiable data removed from analysis.

### 2.4. Statistical Analysis

Kaplan–Meier survival analyses and log-rank tests for univariable analyses were performed using GraphPad Prism version 8.4.1 for Macintosh (GraphPad Software, San Diego, CA, USA) for the prognostic scores detailed in Table 1. Results were reported as median and range. Multivariable analysis was performed by means of the Cox proportional hazards regression model using IBM SPSS Statistics for Macintosh, Version 26.0 (IBM Corp, New York, NY, USA). Only prognostic scores that were significant (*p* < 0.05) on univariable analysis were included in the multivariable analysis. Hazard ratio and 95% confidence intervals (CI) were reported; all statistical tests were two-sided. *p* < 0.05 was considered statistically significant. Where multiple comparisons for univariable analysis were made for the same prognostic score, a Bonferroni correction was applied due to the increased likelihood of type-one error. Adjusted *p* < 0.0167 was regarded as significant.

## 3. Results

### 3.1. Study Cohort

A total of 77 patients with G3-NET/NECs were included from three hospitals. Of these, 32 (41.6%) were G3-NETs and 45 (58.4%) were NECs. The median age of diagnosis of the cohort was 63.4 years (range: 31.1–85.2 years). The most common primary tumour site was the pancreas (23.4%) including one functioning somatostatinoma; followed by the hindgut (colon and rectum, 20.8%); foregut (oesophagus and stomach, 13%); and small bowel (duodenum and jejunum, 5.2%). Furthermore, 37.6% of patients had an unknown primary tumour site. The majority of patients had synchronous metastases at initial diagnosis (84.3%) and the commonest site was the liver (62.3%). The median Ki-67 was 60%. No differences were observed at baseline between G3-NETs and NECs, except for PS with more G3-NETs having a PS of 0 compared to NECs (40.6% vs. 13.3%, *p* = 0.04) (Table 2).

Most patients were treated with at least one line of palliative chemotherapy (81.8%), with the vast majority (93.7%) receiving platinum-based chemotherapy. First-line combinations were predominantly carboplatin-based (57.1%), followed by oxaliplatin-based (19.0%) and cisplatin-based (11.1%). First-line platinum drugs were most commonly combined with either etoposide (63.5%) and capecitabine (27.0%). No statistically significant difference was observed between types of systemic therapy administered or differentiation status of the primary tumour (*p* = 0.4). Only four patients had palliative chemotherapy that was not platinum-based, and were treated with capecitabine and streptozocin. A wide variety of cytotoxic drugs were used in second-line treatment including: irinotecan, topotecan, vincristine, gemcitabine, paclitaxel, epirubicin and docetaxel. Furthermore, 18.2% of patients receiving first-line chemotherapy did not go onto receive second line chemotherapy and were transitioned to best supportive care.

### 3.2. Response to Treatment

The overall response rate (ORR) for first-line chemotherapy was 42.8%; complete (CR) and partial response (PR) was attained by 7.9% and 34.9% of patients respectively. Progressive and stable disease was observed in 34.9% and 22.2% of patients respectively. No significant difference was observed in response to chemotherapy between NETs or NECs (*p* = 0.3). ORR was highest for cisplatin-based therapy (57.2%), followed by 55.6% for carboplatin-based therapy and 16.7% for oxaliplatin-based therapy. At the time of data collection, 72.7% (*n* = 56) of the patients were deceased; the median OS was 13.45 months (range: 0.87–65.37 months). No difference was observed between median OS for NETs (14.0 months (95% CI: 8.5–19.5)) and NECs (12.8 months (95% CI: 8.0–17.6)) (*p* = 0.1).

### 3.3. Exploration of Prognostic Scores

At diagnosis, abnormal albumin and CRP levels were present in 55.3% and 44.7%, respectively; 60.7% of patients had an elevated mGPS (>0) at time of diagnosis. A minority of patients had a PLR ≥ 300 (17.6%) or a NLR ≥ 5 (30.6%). In terms of GI-NEC score, 23.5% were in group A and 8.2% were calculated to be in group B. In terms of relationship between the inflammatory scores and clinico-pathological features, a significant relationship was observed between abnormal mGPS and PS ≥ 2 (*p* = 0.001) and raised NLR and PS ≥ 2 (*p* = 0.011). No other relationships were observed, in particular no relationship was observed between inflammatory scores and degree of differentiation or extent of disease.

### 3.4. Inflammatory Scores and Survival

**Figure 1:**
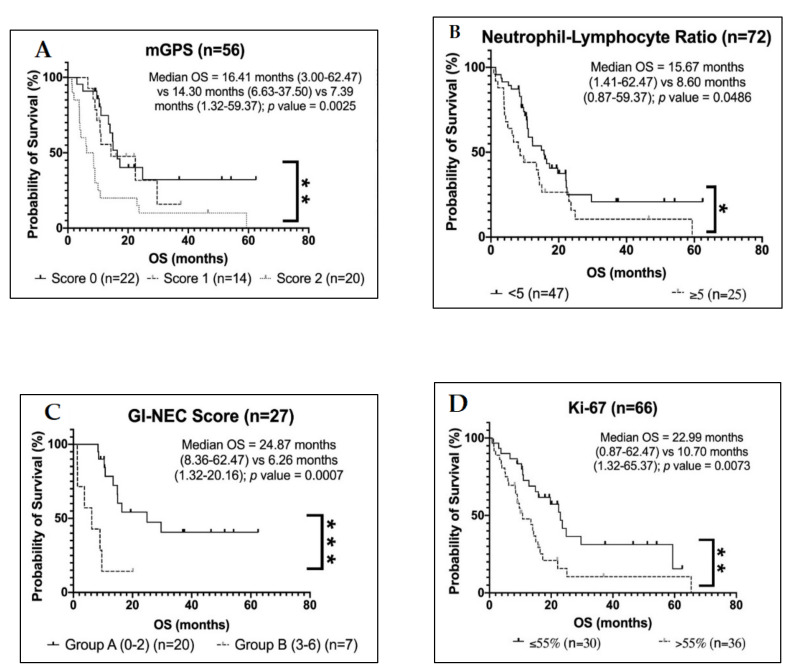
Kaplan–Meier survival curves of OS using different prognostic scores in the entire patient cohort. G3-NETs and NECs were divided into groups defined by different prognostic scores: modified Glasgow prognostic score (mGPS) (**A**); neutrophil-lymphocyte ratio (NLR) (**B**); gastrointestinal neuroendocrine carcinoma (GI-NEC) score (**C**) and Ki-67 using 55% as the cut-off (**D**). Median OS, range and log-rank test *p* value are reported; *p* < 0.05 was regarded as significant. * *p* < 0.05, ** *p* < 0.01, *** *p* < 0.001.

Known predictors of OS including differentiation status, presence of liver metastases and Ki-67 were included in univariable analysis of the entire patient cohort and of these only Ki-67 (*p* = 0.07) was noted to be significant predictor of OS (Table 3). Patients with a mGPS score of 0 had median OS of 16.4 months (95% CI: 13.2–19.7) compared to 14.3 (95% CI: 1.3–27.3) months for mGPS score 1 and 6.3 months (95% CI: 0.7–11.8) for mGPS score 2 (*p* = 0.004) (Figure 1A). Patients with NLR ≥ 5 had a median survival of 8.1 months (95% CI: 4.0–12.1), while patients with NLR < 5 had a median survival of 15.7 months (95% CI: 10.7–20.6) (*p* = 0.04) (Figure 1B). In terms of other staging systems, on univariate analysis, the GI-NEC score (*p* = 0.002) (Figure 1C) was further validated as a significant predictor of survival, such that patients with clinical values placing them into GI-NEC score group B had a four-fold reduction in median OS of 6.3 months (95% CI: 6.7–52.6) compared to a median OS in group A of 29.7 months (95% CI: 0.0–14.0).

Given that there was no significant difference between mGPS 0 and 1 on univariable analysis, these were combined into one subgroup and a comparison was made for mGPS 0/1 vs. 2 for multivariable analysis. On multivariable analysis, mGPS (Hazard Ratio (HR) 4.7, 95% CI: 1.3–16.4; *p* = 0.016), and Ki-67 (HR 5.2, 95% CI: 1.6–16.5; *p* = 0.006) (Figure 1D) remained significant independent predictors of OS in G3-NETs and NECs (Table 3).

We analysed the impact of prognostic scores on the G3-NETs and NEC cohorts independently. When considering G3-NETs on univariable analysis, both PS (*p* = 0.03) and mGPS (*p* = 0.02) were significant predictors of OS. The mGPS score remaining an independent predictor of OS on multivariable analysis (HR 3.4, 95% CI: 1.1–10.3; *p* = 0.03) (Table 4).

Univariable analysis of the NEC cohort, identified PS (*p* < 0.001), Ki-67 > 55% (*p* = 0.001), mGPS (*p* = 0.04) and the GI-NEC score (*p* = 0.007) as significant predictors of OS (Table 5). Of interest, the receipt of platinum was associated with improved OS in this cohort (HR 0.2, 95% CI: 0.1–0.6; *p* = 0.003) (Table 5). On multivariable analysis, advanced PS (HR 4.6, 95% CI: 1.5–14.3; *p* = 0.009) and Ki-67 > 55% (HR 10.33, 95% CI: 2.5–41.9; *p* = 0.001) remained independent predictors of OS. A trend was observed between improved OS and receipt of platinum-based chemotherapy (*p* = 0.05).

## 4. Discussion

There is considerable heterogeneity in the reported prognosis in patients with G3-NET/NECs, ranging from 5 to 34 months [3,22]. There is a need for further stratification of prognosis in the G3 subgroup of NENs, a concept gaining increasing traction with the recognition of the impact of tumour differentiation on response to treatment and survival outcomes [3]. As sustained inflammation acts as one of the principal factors thought to promote the development of neoplasia, we compared the utility of three, widely used inflammation-based prognostic scores in determining overall survival in this heterogeneous patient group [23]. To our knowledge, the prognostic performance of these tests has never previously been studied in a comparative fashion in patients with G3-NETs or NECs. We also explored the prognostic ability of these scores compared with the GI-NEC score and Ki-67. On multivariable analysis of the entire cohort, the mGPS and Ki-67 were found to be independent prognostic markers of OS. When considering G3-NETs, mGPS retained independent prognostic ability but this not the case in patients with NECs in which PS and Ki-67 remained as the only independent prognostic markers.

Whilst the role of inflammation has been studied in NENs, no one study has explored the prognostic ability specific to high grade NENs [24]. Zou et al. [16] explored the prognostic ability of a number of inflammation-based indices in 135 patients with advanced or metastatic NENs. They noted that the high-sensitivity inflammation-based prognostic index, a composite of CRP and WCC, was increased in patients with high grade tumours and was predictive of prognosis, further lending support for the prognostic role of inflammation in NECs. The same paper did not find NLR or PLR to be independently prognostic in patients with NENs, which may relate in part to the use of median NLR and PLR as cut-off values. Similarly, Gaitanidis et al. utilised the median NLR and PLR to predict PFS and recurrence in 97 patients with pNENs undergoing surgical resection [25] and reported that whilst PLR was associated with the presence of metastases, neither were independent predictors of survival. We used similar cut-off values for PLR (≥ 300 × 10^9^/L) [18] and NLR (≥ 5 × 10^9^/L) [21] but were unable to establish either as an independent prognostic index.

Whilst mGPS was significant on multivariable analysis, no significant differences in predicting OS was observed between patients with mGPS = 1 and mGPS = 0. The lack of difference may be due to the small sample size, and these findings should be explored further in a larger cohort. Conflictingly, Zou et al. [16] did not find mGPS to be an independent prognostic marker. As our study was limited only to patients with G3-NETs and NECs, we suggest the prognostic ability of mGPS may be grade-dependent in NENs.

Ki-67 is well established as a prognostic marker and its independent prognostic significance was expected [26]. However, Ki-67 is not a dynamic marker of tumour behaviour as it necessitates repeat biopsy. It is well recognised that the biologic behaviour of NENs can change over time as tumours de-differentiate. Repeat biopsies are not without risk to the patient and lack patient acceptability. Moreover, tumour heterogeneity cannot be fully assessed by biopsy as only a small part of the tumour is assessed. Circulating prognostic markers are therefore attractive in their non-invasive nature and ease of use. Lamarca et al. [14] reported that a Ki-67 of 80% is an important threshold between prognostic groups on receiver operating characteristic (ROC) curve analysis. However, the NORDIC-NEC study ROC analysis suggested that the Ki-67 cut-off of 55% was the most informative with respect to treatment response and prognosis, a position supported by European Neuroendocrine Tumour Society and World Health Organisation (WHO) guidelines [3]. Previous work illustrated that patients with NECs with a Ki-67 ≥ 55% respond better to chemotherapy than patients with Ki-67 < 55%, but are reported to have worse OS than Ki-67 < 55%, results supported by our findings [3].

The GI-NEC score designed by Lamarca et al. [14] combines five prognostic markers: ALP, LDH, Ki-67, ECOG-PS and presence of liver metastases and was validated in NECs. They identified two groups with distinct prognoses with Group A having a median OS of 19.4 months compared to a median OS of 5.2 months in Group B. Concordantly, in our study, risk of death was four times greater for patients in group A compared to group B on univariable analysis. A key limitation of the GI-NEC score is the incorporation of ECOG-PS. Although ECOG-PS is commonly used in clinical practice, it is a subjective assessment of patients’ experience of symptom-burden and therefore is susceptible to bias [27]. Failure to identify the GI-NEC score as an independent prognostic factor in our study is likely to be due to the small number of patients in our study with a GI-NEC score resulting in an imbalance in numbers for each group and therefore a type-two error. Consistent with the GI-NEC study and the NORDIC NEC study, we identified PS as being an independent prognostic factor, which further supports the published findings [3,14].

Systemic inflammation is a recognised feature of cancer development, progression and prognosis [28]. However, the mechanisms underlying cancer-related inflammation remain to be fully elucidated. Highly proliferative tumours, such as NECs, are thought to either outgrow their blood supply causing hypoxia and necrosis or stimulate increased cytokine production, which draws immune cells to the tumour site. Circulating cytokines and immune cells produce a systemic inflammatory response, reflected by increases in acute-phase proteins such as CRP, albumin and LDH. The tumour-derived cytokine IL-6, which is associated with high circulating levels of CRP, have been shown to be prognostic in pancreatic NENs [28,29], lending support to a CRP-based inflammatory score in NENs. Although tumour-derived cytokines such as IL-1*β*, IL-6 and the T-cell derived cytokine TNF-*α* are implicated in the systemic inflammatory response [28,30], no one study has found an association between individual cytokine levels and any of the inflammatory scores, suggesting a complex interplay of the inflammatory response.

The current WHO classification recognises the heterogeneity in clinical course and response to treatment in grade 3 NENs, and sub-divides this grade in an effort to improve understanding of the natural progression of this diagnosis and treatment recommendations. Many of the patients recruited to this study started treatment prior to the current WHO definition of grade 3 NETs and hence were treated with systemic chemotherapy initially. This real-world dataset enhances the data available regarding the treatment response of G3-NETs and NECs to chemotherapy. Of interest, we did not observe a difference in survival between either group, which may be attributed to both sample size and potential selection bias.

In addition to the aforementioned limitations, our work is subject to the inherent restrictions of retrospective studies. Our results were influenced by selection bias with regards to our patient population and inconsistent record keeping between centres. Additionally, due to the rarity of G3-NETs and NECs, our sample size was limited and comparative group numbers were uneven. Thus, statistical analyses may have been underpowered and influenced by potential type-two errors. A prospective study design with a larger cohort would minimise this risk. These limitations should be mitigated in further work.

Nonetheless, our study identified mGPS as an independent predictor of survival in patients with G3-NETs and NECs arising from a wide range of primary sites. This contributes to the growing evidence-base for a score to accurately identify NEC patients with better prognoses who are more likely to benefit from active treatment. Prospective validation in a large cohort of G3-NEC patients is required to ensure results are reproducible. This score could be used to recruit patients for clinical trials, such as the NET-02 [31], where reducing variability within the patient population is beneficial. Our study adds to the evidence-base clinicians may refer to when considering prognosis as a factor for recommending treatment options in patients with NECs. mGPS is derived from routinely assessed haematological parameters and can easily be implemented into clinical practice.

## 5. Conclusions

In a real-world study of G3-NETs and NECs, we have illustrated the utility of inflammatory-based prognostic tools for survival. These warrant further validation in larger studies.

## Figures and Tables

**Table 1:** List of prognostic scores and the defined comparative groups.

Prognostic Score	Comparative Groups
Neutrophil-Lymphocyte Ratio [21]	Ratio < 5
	Ratio ≥ 5
Platelet-Lymphocyte Ratio [18]	Ratio < 300
	Ratio ≥ 300
Modified Glasgow Prognostic Score (mGPS) [17]	Score 0 = CRP ≤ 10 mg/L + Albumin < 35 g/L
	Score 0 = CRP ≤ 10 mg/L + Albumin ≥ 35 g/L
	Score 1 = CRP > 10 mg/L + Albumin ≥ 35 g/L
	Score 2 = CRP > 10 mg/L + Albumin < 35 g/L
Gastrointestinal Neuroendocrine Carcinoma (GI-NEC) Score [14]	Group A = 0–2 points
	Group B = 3–6 points

Abbreviations: C-Reactive protein (CRP), white blood cell (WBC). Points for the GI-NEC score are allocated for: presence of liver metastases (1), alkaline phosphatase (0 = ≤82, 1 = 83–289, 2 = ≥290 U/L), lactate dehydrogenase (0 = ≤827, 1 = ≥828 U/L), Performance status (PS) (0 = 0/1, 1 = ≥2), Ki-67 (0 = ≤80, 1 = >80%) [14].

**Table 2:** Baseline characteristics and biochemical results of patient cohort.

Variable	Overall Cohort (*n* = 77) No. (%)	G3-NETs (*n* = 32)	NECs (*n* = 45)	*p*-Value
Median Age (IQR (Interquartile range)), years	63.1 (22.1)	63.4 (22.2)	62.6 (23.2)	0.7
Sex				
Male	36 (46.8)	17 (53.1)	19 (41.2)	0.2
Female	41 (53.2)	15 (46.9)	26 (57.8)	
Stage				
Locally advanced	9 (11.7)	7 (77.8)	2 (22.2)	0.02
Metastatic	68 (88.3)	25 (36.8)	43 (63.2)	
Site of primary tumour				
Small bowel	4 (5.2)	4 (5.2)	4 (5.2)	0.012
Stomach	5 (6.5)	0 (0)	5 (11.1)	
Oesophagus	5 (6.5)	1 (3.1)	4 (8.9)	
Large bowel	15 (19.5)	8 (25.0)	7 (15.6)	
Pancreas	18 (23.4)	9 (28.1)	9 (20.0)	
Unknown primary	17 (22.1)	3 (9.4)	14 (31.1)	
Other	13 (16.9)	7 (21.9)	6 (13.3)	
Liver Metastases present (yes)	48 (62.3)	17 (53.1)	31 (68.9)	0.1
Number of Metastatic Sites				
<1	42 (54.5)	15 (46.9)	17 (53.1)	0.2
≥2	35 (45.5)	27 (60.0)	18 (40.0)	
Median Ki-67 (%) (IQR) (*N* = 69)	60 (57)	72.5 (59)	50 (47)	0.3
Median Neutrophil count (IQR), ×10^9^/L (*n* = 74)	6.1 (6.1)	5.7 (4.5)	6.9 (6.1)	0.1
Median Lymphocyte count (IQR), ×10^9^/L (*n* = 74)	1.5 (1.0)	1.6 (0.5)	1.4 (0.9)	0.8
Median WBC count (IQR), ×10^9^/L (*n* = 75)	8.9 (6.6)	7.7 (5.6)	9.5 (6.7)	0.2
Median Platelet count (IQR), ×10^9^/L(*n* = 75)	311.5 (129)	282.0 (127.0)	316 (165)	0.1
Median CRP (range), mg/L (*n* = 58)	21.8 (51.4)	17.0 (36.4)	38.0 (77.0)	0.2
Median Albumin (range), g/L (*n* = 75)	36 (11)	38 (13)	36 (11)	0.2
Median ALP (range), U/L (*n* = 74)	113.5 (119)	90.0 (81)	114.5 (99.0)	0.08
Median LDH (range), U/L (*n* = 28)	205 (107)	185 (280)	217 (81)	0.3
CgA (IQR)	51 (173)	44.0 (203)	37.5 (48)	0.4
ECOG-PS				
0	19 (24.7)	13 (40.6)	6 (13.3)	0.04
1	27 (35.1)	7 (21.9)	20 (44.4)	
≥2	31 (40.2)	12 (37.5)	19 (42.2)	
Received Palliative Chemotherapy (yes)	63 (81.8)	26 (81.3)	37 (82.2)	0.6
Received Platinum-based Chemotherapy (yes)	59 (76.6)	22 (73.3)	33 (78.6)	0.4

Received platinum chemotherapy = first or second-line treatment included a platinum drug. Abbreviations and normal ranges for laboratory results are as follows: Neutrophil count 2.0–7.1 × 10^9^/L; Lymphocyte count 1.1–3.6 × 10^9^/L; White Blood Cell (WBC) count 4.2–11.2 × 10^9^/L; Platelet count 135–400 × 10^9^/L; C-Reactive Protein (CRP) < 5 mg/L; Albumin 35–50 g/L; Alkaline phosphatase (ALP) 30–130 U/L; Lactate dehydrogenase (LDH) 125–243 U/L.

**Table 3:** Univariable and multivariable analysis for overall survival (entire cohort).

Prognostic Score	Univariable Analysis		Multivariable Analysis	
	Hazard Ratio (95% CI)	*p*	Hazard Ratio (95% CI)	*p*
Differentiation status	1.6 (0.9–2.9)	0.1		
PS ≥ 2 vs. <2	3.9 (2.2–7.0)	**<0.001**	3.3 (0.9–11.5)	0.06
Ki-67, ≤55% vs. >55%	2.3 (1.3–4.3)	**0.006**	5.2 (1.6–16.5)	**0.006**
Presence of Liver Metastases	1.4 (0.8 to 2.4)	0.2		
Received Platinum Chemotherapy	0.9 (0.5–1.9)	0.8		
Neutrophil-Lymphocyte Ratio (≥5 vs. <5)	1.8 (1.0–3.1)	**0.04**	1.2 (0.4–3.5)	0.8
Platelet-Lymphocyte Ratio (≥200 vs. <200)	1.2 (0.6–2.2)	0.6		
mGPS	-	**0.003**	4.7 (1.3–16.4)	**0.016**
Score 0 vs. 1	1.2 (0.5–2.9)	0.6		
Score 0 vs. 2	3.0 (1.4–6.3)	**0.003**		
GI-NEC Score (B vs. A)	4.7 (1.6–13.7)	**0.004**	1.6 (0.3–7.9)	0.6

*p* < 0.05 was regarded as significant. Statistically significant results were highlighted in **bold**.

**Table 4:** Univariable and multivariable analysis for overall survival in G3-NETs.

Prognostic Score	Univariable Analysis		Multivariable Analysis	
	Hazard Ratio (95% CI)	*p*	Hazard Ratio (95% CI)	*p*
PS ≥ 2 vs. <2	3.3 (1.1–9.4)	**0.03**	2.2 (0.7–6.8)	0.2
Ki-67, ≤55% vs. >55%	1.9 (0.7–5.5)	0.2		
Presence of Liver Metastases	1.1 (0.4–3.1)	0.8		
Received Platinum Chemotherapy	2.9 (0.6–12.8)	0.2		
Neutrophil-Lymphocyte Ratio (≥5 vs. <5)	1.8 (0.6–5.0)	0.3		
Platelet-Lymphocyte Ratio (≥200 vs. <200)	1.6 (0.4–5.6)	0.5		
mGPS (2 vs. 0/1)	3.6 (1.2–10.8)	**0.02**	3.4 (1.1–10.3)	**0.03**

*p* < 0.05 was regarded as significant. Statistically significant results were highlighted in **bold**.

**Table 5:** Univariable and multivariable analysis for overall survival in NECs.

Prognostic Score	Univariable Analysis		Multivariable Analysis	
	Hazard Ratio (95% CI)	*p*	Hazard Ratio (95% CI)	*p*
PS ≥ 2 vs. <2	3.8 (1.9–7.4)	**<0.001**	4.6 (1.5–14.3)	**0.009**
Ki-67, ≤55% vs. >55%	5.2 (2.1–13.1)	**0.001**	10.2 (2.5–41.9)	**0.001**
Presence of Liver Metastases	1.4 (0.7–2.8)	0.3		
Received Platinum Chemotherapy	0.2 (0.1–0.6)	**0.003**	0.2 (0.05–1.0)	0.05
Neutrophil-Lymphocyte Ratio (≥5 vs. <5)	1.6 (0.8–3.2)	0.2		
Platelet-Lymphocyte Ratio (≥200 vs. <200)	1.1 (0.6–2.4)	0.7		
mGPS (2 vs. 0/1)	2.3 (1.0–4.9)	**0.04**	2.5 (0.9–7.2)	0.09
GI-NEC Score (B vs. A)	6.3 (1.6–24.2)	**0.007**	-	-

*p* < 0.05 was regarded as significant. Statistically significant results were highlighted in **bold**. Unable to generate HR for GI-NEC on multivariable analysis due to large amount of missing data.

## Data Availability

Data is available from the corresponding author after appropriate review.
